# Investigation of the Effect of Structural Properties of a Vertically Standing CNT Cold Cathode on Electron Beam Brightness and Resolution of Secondary Electron Images

**DOI:** 10.3390/nano11081918

**Published:** 2021-07-26

**Authors:** Ha Rim Lee, Da Woon Kim, Alfi Rodiansyah, Boklae Cho, Joonwon Lim, Kyu Chang Park

**Affiliations:** 1 Department of Information Display, Kyung Hee University, Dongdaemun-gu, Seoul 024471, Korea; hrim@khu.ac.kr (H.R.L.); dawoon0601@khu.ac.kr (D.W.K.); rodiansyahalfi@khu.ac.kr (A.R.); 2 Advanced Instrumentation Institute, Korea Research Institute of Standards and Science (KRISS), 267 Gajeong-ro, Yuseong-gu, Daejeon 34113, Korea; blcho@kriss.re.kr

**Keywords:** carbon nanotube, field emission, electron beam, electron microscope

## Abstract

Carbon nanotube (CNT)-based cold cathodes are promising sources of field emission electrons for advanced electron devices, particularly for ultra-high-resolution imaging systems, due to their high brightness and low energy spread. While the electron field emission properties of single-tip CNT cathodes have been intensively studied in the last few decades, a systematic study of the influencing factors on the electron beam properties of CNT cold cathodes and the resolution of the secondary electron images has been overlooked in this field. Here, we have systematically investigated the effect of the structural properties of a CNT cold cathode on the electron beam properties and resolution of secondary electron microscope (SEM) images. The aspect ratio (geometric factor) and the diameter of the tip of a vertically standing CNT cold cathode significantly affect the electron beam properties, including the beam size and brightness, and consequently determine the resolution of the secondary electron images obtained by SEM systems equipped with a CNT cold cathode module. Theoretical simulation elucidated the dependence of the structural features of CNT cold cathodes and electron beam properties on the contribution of edge-emitted electrons to the total field emission current. Investigating the correlations between the structural properties of CNT cold cathodes, the properties of the emitted electron beams, and the resolution of the secondary electron images captured by SEM equipped with CNT cold cathode modules is highly important and informative as a basic model.

## 1. Introduction

Field emission electron sources have attracted enormous research attention in the field of electron emission and electron devices, due to their inherent merits, including high brightness, coherence, and low energy spread [1,2,3,4,5]. Owing to those advantages, distinguished from conventional thermionic electron sources, field emission electron sources, i.e., cold cathodes, have been considered as a promising component for advanced microscope systems, enabling ultra-high-resolution imaging at the nano- or atomic scales [6,7,8]. As the best choice for cold cathode materials, carbon nanotubes (CNTs) have drawn much research attention due to their highly efficient electron field emission properties, originating from the synergistic effect of high electrical conductivity and high aspect ratio [9,10,11,12,13,14]. Especially, the small radius of curvature of the tip of CNT cold cathodes induces a substantial field enhancement effect and decreases the operating voltage of the field emission devices. It is beneficial for a miniaturized electron emission source to be able to be driven stably up to few micro-amperes of current [15,16,17,18,19,20]. Moreover, mature CNT synthesis techniques have achieved uniform large areal arrays and site-selective vertically aligned structures. Despite the suitable features of CNTs for high-performance cold cathodes, there have been difficulties in realizing CNT cold cathodes for ultra-high-resolution microscope systems due to the lack of a systematic study on the correlation between the imaging resolution and the properties of electron beams emitted by such cathodes.

Ultra-high-resolution imaging systems using electron beams require two vital conditions: high electron emission current and small enough probe size [21]. In this regard, it is necessary to investigate the electron emission current and electron probe size of electron beams generated from CNT cold cathodes in order to realize CNT cold cathode-based ultra-high-resolution imaging systems. It is especially important and valuable to study vertically aligned individual CNT cold cathodes as a basic model to develop sophisticated CNT cold cathodes. Even though a CNT cold cathode effectively enhances the electric field at the tip of the one-strand electron field emitter by removing the screening effect, it is usually unstable with high field emission current value or long-term electron emission [22]. This leads to a lack of systematic and reliable studies on the electron emission current and probe size of individual CNT emitters. Moreover, precise control of the location and reproducible shape engineering of CNT cold cathode are also required to guarantee reliability.

In this work, we studied the effects of the structural properties of CNT cold cathodes on the electron beam properties and the resolution of secondary electron images. CNT cold cathodes were grown on silicon substrates *via* plasma-enhanced chemical vapor deposition (PECVD) with patterned Ni catalysts, offering precise control of the location of vertically aligned CNTs. The aspect ratio and the tip diameter of the CNT cold cathode significantly affects the diameter and brightness of the electron beam. CNT cold cathodes show high field emission current, high beam brightness, and small beam diameter with increasing aspect ratio (high geometric factor, β_geo_) and decreasing tip diameter. In theoretical study, a simulated electron trajectory consistent with empirical results indicates that the β_geo_ and the tip diameter of the CNT dominate the size and areal uniformity of the resultant electron beam. Consequently, the resolution of images obtained with a CNT cold cathode module mounted in a scanning electron microscope (SEM) system improves with high β_geo_ and small tip diameter.

## 2. Materials and Methods

The used CNT cold cathode was composed of a vertically standing, one-strand and cone-shaped CNTs grown on a highly doped n-type silicon (100) substrate. The location of a CNT cold cathode was controlled to be in the center of the Si substrate, cut into a 4 × 4 mm^2^ piece. The structure of the emitters was determined by the growing conditions. A nickel dot, 3–5 μm in diameter and fabricated by a conventional photolithography process, determined the position of the CNT cold cathode. The advantage of CNT field emitters is that the number of emitters can be precisely adjusted, allowing control of the electron emission current value by adjusting the number of CNT field emitters [23].

**Figure 1:**
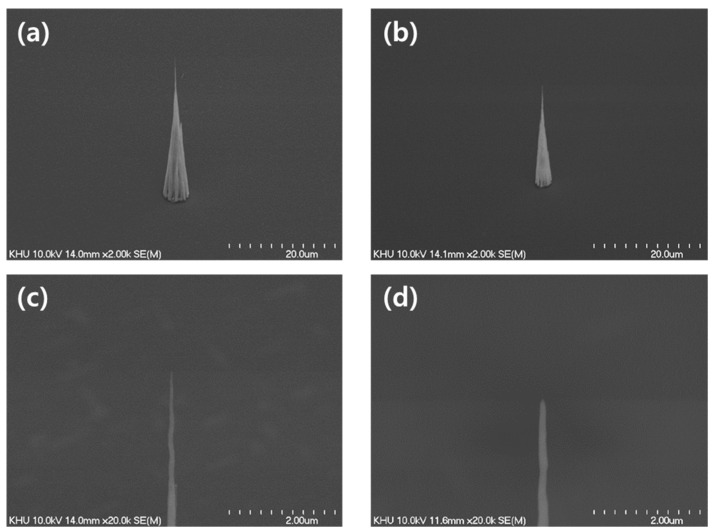
SEM images of CNT cold cathodes: (**a**) geometric factor 1560 (1560-CNT) and (**b**) 550-CNT cold cathode. (**c**) Magnified image of (**a**) at tip region; (**d**) magnified image of (**b**).

The location of the CNT cold cathode on the Si substrate was controlled by the predetermined locations of 20 nm-thick Ni catalysts, using a photolithography technique. The prepared Ni patterns were annealed to produce seeds for the growth of CNTs [24,25]. The CNT cold cathodes were grown using a triode-type, direct current, plasma-enhanced chemical vapor deposition (DC-PECVD) system. The emitter grown by PECVD is characterized by a cone shape, as shown in Figure 1. The CNT cold cathodes grows from several nano-sized nickel grains and merges into one at the tip end [26]. After the forming process, CNTs are grown in the DC-PECVD system, and the emitter structure can be adjusted according to the growing conditions. Several parameters determine the structure of a CNT cold cathode: seed size, temperature, pressure, plasma current, and gas ratio [27]. These growing conditions are listed in Table 1. Under such conditions, we can grow CNTs by determining the range of β_geo_ of CNT cold cathodes.

Figure 1 shows scanning electron microscope (SEM) analysis of CNT cold cathodes with two β_geo_ values, 1560 and 550. The geometric field enhancement factor is determined by the apex tip radius and height, and is defined as the height of the emitter divided by the radius of the tip [14,28]. A 1560 emitter has a height of 39 μm and a tip radius of 25 nm, and a 550 emitter has a height and radius of 27.5 μm and 75 nm. Based on those parameters, we modeled the SOURCE 2D simulation (Munro’s Electron Beam Software, London, UK) to analyze the characteristics of the trajectory, the emission current, and the brightness of the electron beam. The SOURCE 2D simulation was calculated, taking into account the space charge effect using the second order finite element method [29].

The evaluation of the electron emission properties was conducted in diode mode, with a homemade phosphor screen, under high vacuum level (<10^−7^ torr). We carried out comparative analyses of the electron emission characteristics and emission patterns in the diode structure with several β_geo_ values. The distance from CNT cold cathode to anode was 250 μm.

To observe the structure of CNT cold cathodes, SEM analysis was conducted (Hitachi S-4700). We used a DC power supply (Spellman SL1200, Keithley 248) and multimeter (Agilent 34401A, Keithely 6485) to measure the field emission characteristics. The electron emission characteristics were analyzed using the Fowler–Nordheim (F–N) theory. The electron emission pattern was observed by optical microscope.

## 3. Results and Discussion

**Figure 2:**
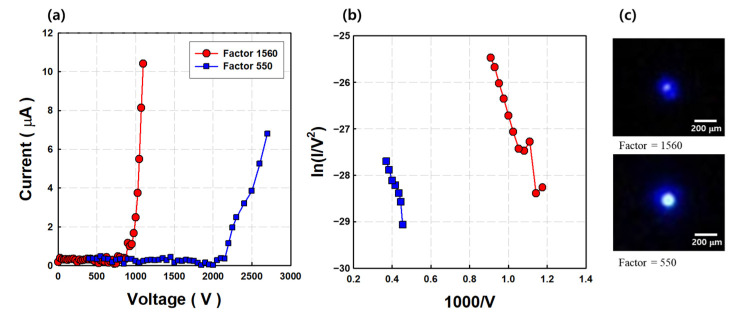
Comparison of field emission properties. (**a**) Field emission properties; (**b**) F–N plot; (**c**) captured image of field emission patterns on phosphor screen at 1 μA of emission current.

The basic field emission characteristics were measured in the diode structure with the phosphor anode for evaluation of the CNT cold cathode. Figure 2a,b show comparisons of the field emission characteristics and FN plots according to β_geo_ values, respectively. The CNT field emitters with β_geo_ values of 1560 and 550 showed 1 μA of emission current at 950 and 2250 V, respectively. The slope of the FN plot of the 1560 CNT cold cathode was −35006, and the slope of the 560 was −48725 in the linear region of Figure 2b. The CNT emitter with high β_geo_ showed high emission current at lower voltages. The difference in the field emission properties is attributed to the lower threshold voltage at the edge of the emitter tip. It indicates that the enhancement of the applied electrical field is facilitated by the CNT with small tip size, compared with the CNT with large tip size. Also, the height of the emitter itself is an important parameter. Therefore, many studies refer to a high aspect ratio (β_geo_) as a very important variable. Figure 2c shows a comparison of electron emission patterns at a current of 1 μA. The CNT field emitters with β_geo_ values of 1560 and 550 show 55 μm of the beam size at 950 V and 114 μm at 2250 V, respectively.

The source of electrons for an electron microscope can be evaluated by characterizing simple electron emission, including the solid angle, the virtual source size, and the brightness of the electron beam. In our previous study, we studied reduced brightness (*B_r_*) calculation using electron emission patterns to evaluate CNT cold cathodes [30]. *B_r_* measures the spot size and the amount of emission current that can be concentrated at a particular solid angle. It is a function of the virtual radius source size, the brightest part of the emitted electron beam, and the current densities corresponding to the beam potential [31]. The virtual source radius is the area where electrons are generated. The high brightness means that the electrons are emitted at a narrow solid angle, the size of the starting beam trajectory is small, and there is a large amount of emission current at low voltage. Thus, B_r_ is expressed by the following equation [32], where $J_{\Omega }$ is the angular current density and $r_{v}$ is the virtual source radius:

(1) $$B_{r}=\frac{J_{\Omega }}{\pi r_{v}{}^{2}V}.$$

The electron beam pattern was observed by optical microscope and was measured with an image analysis tool to obtain the correct beam size. Beam size values of 55 μm and 114 μm were obtained from the full width at half maximum (FWHM) value at the bright spot using Gaussian four-peak parameter fitting [33]. As a result of emission patterns, the solid angles of 1560- and 550-CNT emitters were 0.053 and 0.2 sr, respectively, at an emission current of 1 μA. To calculate the virtual source radius, we refer to Fowler–Nordheim (F–N) theory, which understands the field emission phenomenon mathematically. Its emission current (J) density is as follows [34]:

(2) $$J=1.54\times 10^{-6}\frac{F^{2}}{\emptyset }exp\left(-6.83\times 10^{-7}\frac{\emptyset^{3/2}}{F}\right)$$

(3) F=βV

(4) $$\beta_{geo}=m+h/R$$

where F and ∅ are the local electric field and the work function in electron volts, respectively. R is the radius of curvature, h is the height of the emitter, and m is a variously taken constant of 0, 2, or 3; m converges to 0 if the height of the emitter is considerably greater than the curvature radius (h >> R). This $\beta_{geo}$ calculation method was first proposed by Vibrans (1964) [35]. As indicated in the F–N equation, two parameters affect the field emission properties of a field emitter. The work function of CNTs is widely known to be 5 eV [36,37]. These two variable values are reflected in the tunneling parameters (d) and are important factors in calculating the virtual source radius [38]:

(5) $$d=1.54\times 10^{-6}\frac{ehF}{4\pi \sqrt{2m\emptyset }\times t\left(y\right)}$$

(6) $$t\left(y\right)=1+0.1107y^{1.33}$$

(7) $$r_{v}=\sqrt{\frac{3Rd}{ekF}}$$

where t(y) and y are slowly varying functions of F and ∅ [39]. The electric field applied to the CNT cold cathode depends on the structure of the emitter (β_geo_). The tunneling parameters of the CNT emitter range from 0.1 to 0.3 eV. The $r_{v}$ of the spherical cap structure is determined by d, the physical source diameter of field emitter and the local electric field, and k is the field enhancement related factor, which frequently takes a value of five [40]. The physical source diameter (R) of the CNT cold cathode was 50 and 150 nm, as shown in Figure 1, and we could calculate that $r_{v}$ was 0.8 and 1.3 nm for the CNT cold cathode, respectively. As a result, the theoretical angular current density of the 1560- CNT cold cathode was 20.89 μA·sr^−1^ and *B_r_* was 1.08 × 10^10^ A·m^−^^2^·sr^−1^·V^−1^ at an applied voltage of 950 V. For the 550- CNT cold cathode, *B_r_* was 3.7 × 10^8^ A·m^−^^2^·sr^−1^·V^−1^ at an applied voltage of 2250 V and the angular current density was 5.09 μA·sr^−1^. The smaller the value of β_geo_, the higher the required driving voltage, resulting in a larger beam diameter and wide angular current density.

**Figure 3:**
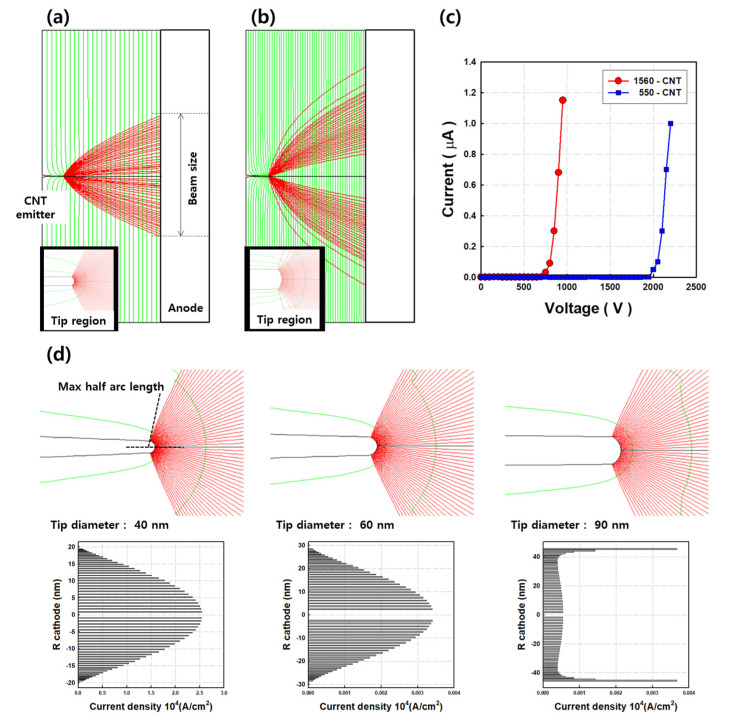
SOURCE 2D simulation results. (**a**) The CNT cold cathode with a geometric factor of 1560. (**b**) The CNT cold cathodes with a geometric factor of 550. (**c**) Comparison of simulated I-V curve. (**d**) Emission current density at tip region with tip diameter.

SOURCE 2D simulation is a useful tool with which to analyze and design electron sources. This tool is available for all types of electron sources, from point cold cathodes to high-current piercing. The simulation study calculates the space charge distribution based on the Poisson equation, and electron trajectories are computed by direct ray tracing [28]. Figure 3a,b shows the beam trajectories of the CNT cold cathodes with 1560 and 550 β_geo_. The figure inset images show the computed electron emission at the tip region. The basic variable parameters are set to the same values, including CNT work function, energy spread, and temperature. The comparative simulation results are highly dependent on the structure of the field emitter. Based on the measured field emission characteristics, the trajectory and beam brightness were computed at an emission current of 1 μA. The distance between source plane and anode was 200 μm and the maximum beam size was 258 μm (Figure 3a) and 381 μm (Figure 3b), respectively. Figure 3c shows the computed I-V curve based on the experimental values. The SOURCE 2D simulation is computed for the launch point of electron emission at the tip region, which is named the maximum half arc length, as shown in Figure 3d (dashed black line in the first image). The maximum half arc length of the 1560- CNT cold cathode was 48 nm, and the current density at the apex of the tip was the highest, calculated as 1.1 × 10^4^ A·cm^–2^. For the 550 emitter, the maximum half arc length was 110 nm and the current density at the edge of the tip was the highest. Figure 3d shows the beam trajectory and emission current density at the CNT tip with various diameters. As the tip diameter increased, the current density at the edge region was enhanced. This indicates that a small-sized CNT tip is highly beneficial to obtain a well-focused and high-density electron beam.

**Figure 4:**
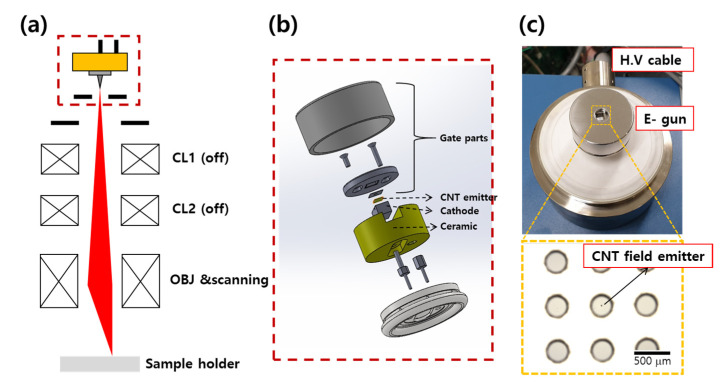
(**a**) Schematic diagram of normal SEM equipment with single CNT emission gun. (**b**) CNT electron gun cartridge. (**c**) Captured image of gun part and alignment with gate electrode and single CNT emitter.

We evaluated the electron emission characteristics and designed an optimized electron beam module based on the protocol reported in our previous study [29]. The CNT cold cathode was applied to an electron microscope imaging device as an electron source. Figure 4a shows the configuration of the system for secondary electron imaging. Typical SEM systems are composed of an electron source, two condenser lenses (CLs), an objective lens (OBJ), and a stage for a sample in a vacuum chamber. The CL serves to demagnify the electron beam and determines the probe size and resolution. The OBJ serves to adjust the working distance. The distance between the electron gun part and OBJ is 400 mm and working distance is 2 mm. Figure 4b shows a schematic illustration and real optical image of the electron beam module with a CNT cold cathode. As shown in real optical images in Figure 4c, the electron beam module consists of CNT cold cathode on the cathode and a mesh-type gate electrode with holes 300 μm in diameter where the CNT cold cathodes are precisely aligned at the center of the aperture. The distance between the gate electrode and the cathode was adjusted to 250 μm, and the anode of the SEM system was located 8 mm below. The lens units and the sample stage in the chamber were connected to the ground. Thus, the electron beam was driven in the range of negative voltage. A wobbling process is generally required to minimize CL aberration, in order to obtain high-resolution images. For precise evaluation of the electron beam characteristics of each CNT cold cathode, secondary electron imaging was carried out with only the as-emitted electron beam from the CNT cold cathode and all CLs turned off.

**Figure 5:**
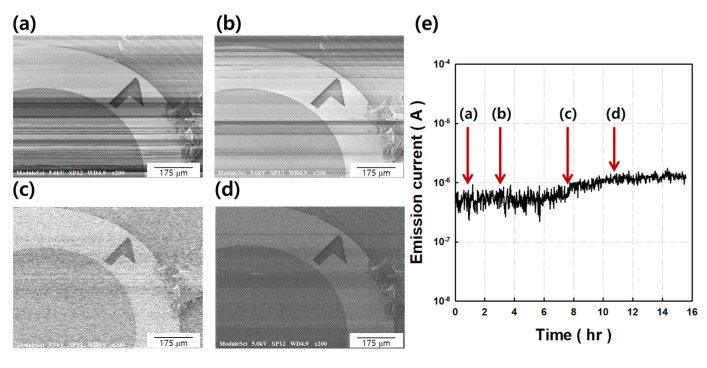
Scanning secondary electron image with CNT cold cathode gun. (**a**–**d**) SEM images of Cu grid mesh (2000) during aging process. (**e**) Electron emission current stability in constant voltage mode (ΔV = 900 V) (Δ V = V_acc_ − V_ex_).

Figure 5a–d shows scanning secondary electron images of copper (Cu) mesh grids (2000) captured during the aging process, obtained by the CNT cold cathode. All SEM images were measured without the CL operation. The electrons were emitted at 10^−8^ torr at the gun part with acceleration voltage (V_acc_) of 5 kV. The resolution of the SEM image was improved with aging time. Figure 5a shows the first image obtained at an emission current of 0.5 μA. The image shows an undesired alternating light and dark line, indicating that the emission current is unstable. Interestingly, the resolution and definition of the measured SEM image increased with aging time. Figure 5e shows the electrical aging process of the CNT cold cathode. The emitter had a β_geo_ of 790 and the relative extraction voltage (ΔV) was 900 V. In the initial 6 h, the average emission current was 0.5 μA in constant voltage mode (ΔV = 900 V), and the current fluctuation was 25%. After 10 h, the average emission current increased to 1 μA, and the current stability was shown to be 15%, which was more stable than the initial one. The stabilized electron emission current yielded improved images, as shown in Figure 5d. It is rationalized with the recovery of defect sites in the CNT cold cathode. After the electrical aging process, the ratio of defect peak to graphite peak increased in the Raman result [41].

**Figure 6:**
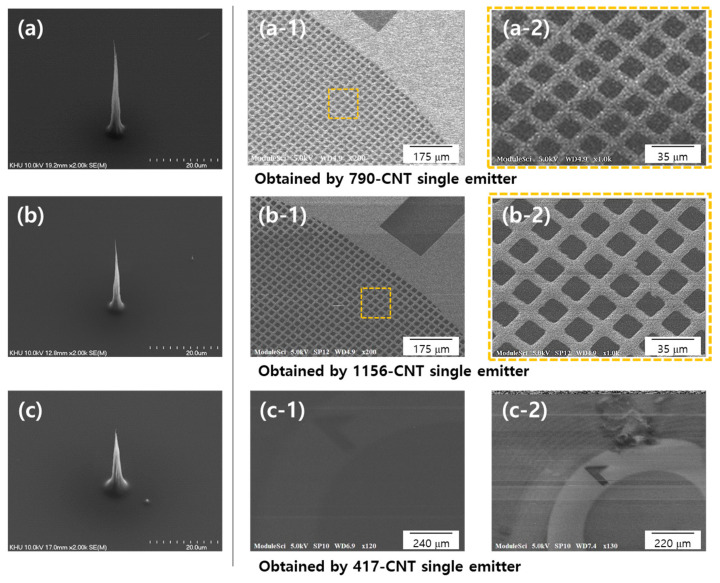
Comparison of scanning secondary electron images with structural properties of CNT cold cathodes. SEM images of a CNT cold cathode with: (**a**) β_geo_ = 790, ΔV = 1200 V; (**b**) β_geo_ = 1560, Δ V = 900 V; and (**c**) β_geo_ = 417, Δ V = 2100 V. (**a-1**–**c-1**) SEM images of Cu grid mesh (2000) at 5 kV acceleration voltage and 1 A emission current. (**a-2**) A 1000× magnified image of dashed square area in (**a-1**). (**b-2**) A 1000× magnified image of dashed square area in (**b-1**). (**c-2**) SEM image obtained at 5 kV and 3 A emission current.

Figure 6 shows SEM images obtained with the CNT cold cathodes with different β_geo_ values; the emitter in Figure 6a had a tip diameter of 100 nm and height of 39.5 μm (β_geo_ = 790), and the one in Figure 6b had a tip diameter of 50 nm and height of 28.9 μm (β_geo_ = 1156). Figure 6(a-1–c-1) show SEM images obtained at an acceleration voltage of 5 kV and 1 μA of emission current. The SEM image in Figure 6a was obtained after the aging process. Figure 6(a-2) shows 1000× magnified SEM images of the region of the dashed square yellow line in Figure 6(a-1). Figure 6(b-1,b-2) shows SEM images obtained with the a CNT cold cathode in Figure 6b under identical conditions as in Figure 6a. Figure 6c shows SEM images obtained with a CNT cold cathode with a tip diameter of 125 nm and height of 26.1 μm (β_geo_ = 417). Despite the higher emission current of 3 μA than in the previous cases, the resulting SEM image was severely blurred, as shown in Figure 6(c-1). Even under three times higher emission current, a high-magnification image could not be properly obtained. This means that the beam brightness is low due to structural limitations.

The electron beam spot size can be calculated using the SEM images by a method proposed by the American Society for Testing and Materials (ASTM) [42]. The beam spot size was calculated using the contrast ratio of the sharp edge of the mesh line in Figure 6(a-2,b-2). The minimum and maximum values were recorded accordingly, and the 20% and 80% values were calculated. The distance between 20% and 80% was obtained using a commercially available image analysis program. The contrast slopes were 43 and 117, respectively. Using this, the electron beam spot size (probe size) was calculated as 0.37 and 0.25 μm, respectively. Relatively large spot size of the electron beam with respect to the virtual source size of 0.8 nm and 1.3 nm can be elucidated with the widespread trajectory of electrons by the change of the equivalent potential line near the tip of a CNT cold cathode and the screening effect among emitted electrons. It can be seen that the CNT cold cathodes with higher aspect ratios have higher brightness, which in turn leads to images with higher resolution. It has been shown that the structural properties of the CNT electron source eventually lead to differences in resolution.

## 4. Conclusions

We investigated the effect of the structural properties of CNT cold cathodes on the electron beam properties and resolution of secondary electron images. The 1560- CNT cold cathode, with a high β_geo_ and small tip diameter, produced a focused electron beam with high brightness and small beam diameter, consequently allowing us to obtain SEM images of high resolution, compared with the SEM images obtained by the 417- CNT cold cathode with a low β_geo_ and large tip diameter. The theoretical study reveals that the contribution of spreading edge-emitted electrons to the total emission current increases with increasing tip diameter. The simulation results rationally explain the limited image resolution obtained by a SEM system equipped with a 417- CNT cold cathode module. Our results indicate that the geometrical factor of the CNT cold cathode is an important influencing factor to obtain ultra-high-resolution images using secondary electron imaging system. 

## Figures and Tables

**Table 1:** Summary of carbon nanotube (CNT) growing conditions.

Geometric Factor	C_2_H_2_:NH_3_ (SCCM)	Voltage (V) (Grid/Substrate)	Pressure (Torr)	Dot Size (μm)	Growing Time (min)
2800–3500	16:160	300/−600	2	3	100
600–1500	16:160	300/−600	3	5	60
~500	16:200	300/−600	2.5	5	90

## Data Availability

The data presented in this study are available on request from the corresponding author.
